# *In Vitro* Antifungal Activity of the Diterpenoid 7α-Hydroxy-8(17)-labden-15-oic Acid and Its Derivatives against *Botrytis cinerea*

**DOI:** 10.3390/molecules14061966

**Published:** 2009-05-26

**Authors:** Leonora Mendoza, Pamela Espinoza, Alejandro Urzua, Marcela Vivanco, Milena Cotoras

**Affiliations:** Facultad de Química y Biología, Universidad de Santiago de Chile, Avenida Bernardo O’Higgins 3363, Santiago, Chile; E-mails: leonora.mendoza@usach.cl (L.M.), pamelaespinozasommer@gmail.com (P.E.); ma.vivanco.m@gmail.com (M.V.); alejandro.urzua@usach.cl (A.U.)

**Keywords:** *Botrytis cinerea*, antifungal compounds, diterpenoids, salvic acid, acetylsalvic acid, *Eupatorium salvia*

## Abstract

We investigated the inhibitory effect of the natural diterpenoids, 7α-hydroxy-8(17)-labden-15-oic acid (salvic acid, **1**), 7α-acetanoyloxy-8(17)-labden-15-oic acid (acetylsalvic acid, **2**) and the hemisynthetic diterpenoids 7α-acyloxy-8(17)-labden-15-oic acids derivatives, 7α-propanoyloxy-8(17)-labden-15-oic acid (propanoylsalvic acid, **3**), 7α-butanoyloxy-8(17)-labden-15-oic acid (butanoylsalvic acid, **4**) and 7α-isopentanoyloxy-8(17)-labden-15-oic acid (isopentanoylsalvic acid, **5**), against *Botrytis cinerea*. Diterpenoid fungitoxicity was assessed using the radial growth test method. All diterpenoids, with the exception of isopentenoylsalvic acid, inhibited the mycelial growth of *B. cinerea* in solid media. Shortest side-chain diterpenoids were more effective than the derivatives with longer chains in the inhibition of *B. cinerea* mycelial growth. The results suggest that hydrophobicity and structural features would be important factors in the antifungal effect of these diterpenoids. Studies on a possible action mechanism of natural diterpenoids, salvic acid and acetylsalvic acid, showed that these diterpenoids exerted their effect by a different mechanism. Salvic acid did not alter cytoplasmic membrane or cause respiratory chain inhibition. Instead, acetylsalvic acid affected the cytoplasmic membrane producing leakage of 260-nm absorbing compounds.

## 1. Introduction

*Botrytis cinerea* is a facultative phytopatogenic fungus that attacks flowers, fruits, leaves, and stems of more than 200 plant species [1]. The continuous use of commercial fungicides such as dicarboximide and benzimidazole has caused the appearance of highly resistant strains of *B. cinerea* and the contamination of soil and water [2]. It has been reported that some natural products isolated from plants exert antifungal activity and that these compounds can be a good alternative to commercial fungicides [3]. Several terpenoids have shown activity against *B. cinerea,* such as the sesquiterpenes 7‑hydroxycalamenene and drimenol, and the diterpenoids kolavenic acid and 2-oxokolavenic acid [4,5]. Studies carried out with diterpenoids kaurenoic acid and 3β-hydroxykaurenoic acid, both isolated from *Pseudognaphalium vira vira,* showed that the hydroxylated diterpenoid is more active against *B. cinerea* than the non-hydroxylated one. 3β-Hydroxykaurenoic acid would produce permeabilization of *B. cinerea* cell membrane [6]. The monoterpenoids thymol and carvacrol also affect the plasmatic membrane of this fungus [7]. Studies in *Candida albicans* have demonstrated that the diterpenoid (*E*)-8 β,17-epoxylabd-12-ene-15, 16-dial produces lysis of its protoplasts [8].

On the other hand, the diterpenoids totarol and 13-epi-sclareol affect the respiratory chain in bacteria [9,10]. Totarol inhibits the oxygen consumption of *Pseudomonas aeruginosa* and the proton translocation in the whole cell [9]. 13-epi-Sclareol affects the oxygen consumption only in Gram-positive bacteria [10]. There are no reports about the effect of diterpenoids on the respiratory chain in fungi.

*Eupatorium salvia* Colla produces a resinous exudate that contains the diterpenoids 7-hydroxy-8(17)-labden-15-oic acid (salvic acid, **1**) and 7α-acetanoyloxy-8(17)-labden-15-oic acid (acetylsalvic acid, **2**). Both compounds show antibacterial activity against Gram-positive bacteria, acetylsalvic acid being the most active. These diterpenoids do not affect Gram-negative bacteria [11]. The activity of these compounds on filamentous fungi has not been reported.

The aim of this study was to analyze the antifungal effect of the natural diterpenoids salvic acid and acetylsalvic acid, and the hemisynthetic derivates propanoylsalvic acid, butanoylsalvic acid, and isopentanoylsalvic acid, against *B. cinerea.* In addition, the effect of natural diterpenoids on oxygen consumption and on membrane integrity was evaluated.

## 2. Results and Discussion

### 2.1. Isolation of natural diterpenoids and characterization of salvic acid

**Figure 1:**
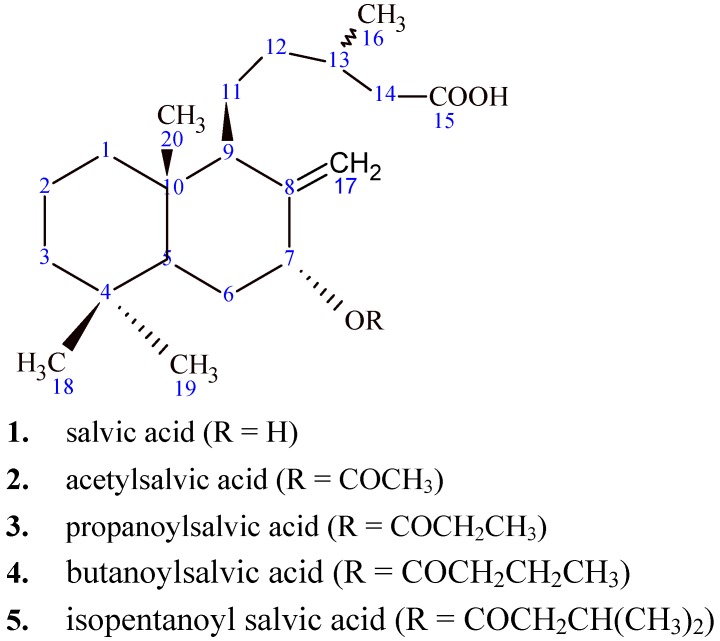
Structure of natural and hemisynthetic diterpenoids.

**Figure 2:**
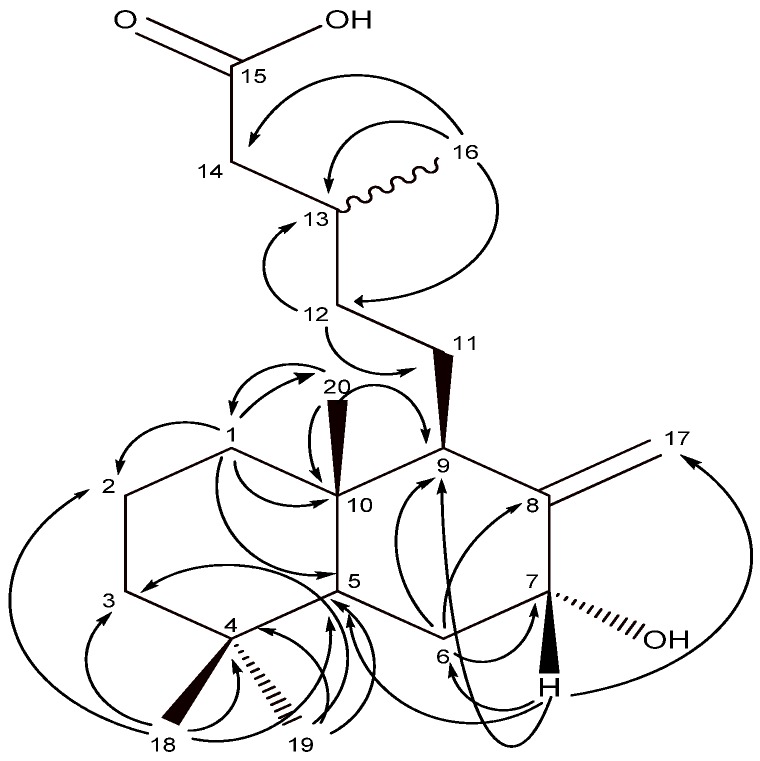
HMBC correlations of salvic acid (**1**).

The compounds used in this study are shown in Figure 1. Salvic acid (**1**) and acetylsalvic acid (**2**) were isolated from the resinous exudates of *E. salvia* leaves. Isolated diterpenoids were identified by comparison of their spectroscopic data (^1^H- and ^13^C-NMR) with those reported in the literature [11,12,13]. Extensive 2D experiments (^1^H-^1^H COSY, HMBC, HSQC and NOESY) allowed complete assignments of ^1^H and ^13^C resonances of salvic acid **1** (Table 1), since only a partial assignment for the structure of this compound was previously reported in the literature [12,13]. From 2D HSQC experiments, the protons showed ^1^*J* connections with the respective carbon atoms. The assignation also was corroborated by 2D HMBC, where protons showed heteronuclear ^2^*J* and ^3^*J* correlations with the carbon atoms (Figure 2).

**Table 1:** ^13^C- and ^1^H-NMR assignment of salvic acid (**1**).

Carbon	^13^C (δ)	^1^H (δ)	Carbon	^13^C (δ)	^1^H (δ)
1	39.00	1.74 (d-t) 1.11 (m)	11	20.80	1.28 (m)
2	19.56	1.51 (m)	12	35.62	1.04 (m) 1.51 (m)
3	42.30	1.31 (m) 1.21 (m)	13	31.11	1.94 (o(a))
4	33.30	-	14	41.34	2.36 (d-d) 2.15 (d-d)
5	47.85	1.59 (m)	15	178.46	-
6	31.08	1.86 (d-d)	16	20.09	0.98 (d)
7	74.37	4.37 (d(a))	17	109.99	5.03 (t(a)) 4.62 (t(a))
8	149.85	-	18	33.50	0.80 (s)
9	51.54	2.07 (d-d)	19	21.72	0.88 (s)
10	40.09	-	20	13.59	0.65 (s)

### 2.2. Antifungal activity characterization

To evaluate the antifungal activity of these natural and hemisynthetic diterpenoids, the effect on the mycelial growth on solid media was determined after 72 hours of incubation (Table 2).

**Table 2:** Effect of salvic acid, acetylsalvic acid and their hemisynthetic derivates on *in vitro* mycelial growth of *B. cinerea*.

Compounds	ED_50_^a^ ± SD (μg/mL)
**1**	53.1 ± 4.6
**2**	60.2 ± 8.4
**3**	59.5 ± 5.5
**4**	100.2 ± 4.9
**5**	> 250
Iprodione	35.0 ± 1.5

^a^Estimation of median effective doses (ED_50_) was based on colony diameter measurements after 72 hours of incubation.

Compounds **1**, **2**, and **3** were the most active. The ED_50_ values were higher to those obtained with the fungicide iprodione used as control.

Diterpenoids with longer side chains presented higher ED_50_ value. Compound **5** presented a very low effect on the mycelial growth of *B. cinerea*. It is important to emphasize that this was an isolate resistant to dicarboximide because ED_50_ is >10 μg/mL. The results showed that the antifungal activity did not differ between compounds with a free hydroxyl group and those with hydroxyl esterified with short carbon chains (compounds **1**, **2** and **3**); therefore, the hydroxyl group does not appear to play an important role in the inhibitory effect of these compounds against *B. cinerea*.

It has been demonstrated that the hydrophobicity of the compounds is a very important factor for fungicidal activity [14,15,16,17]. Therefore, the relationship between the antifungal activity, the lipophilicity (log *P*) and the molecular volume (V) was determined. The values of lipophilicity and molecular volume of the diterpenoids are shown in Table 3. 

**Table 3:** Volume and log *P* values of the diterpenoids.

Compounds	Volume (Å^3^)	Log *P*
****1****	974	4.56
****2****	1069	4.69
****3****	1136	5.32
****4****	1190	5.72
****5****	1219	6.05

The results show that diterpenoids with small and lipophilic groups (compounds **1**, **2** and **3**) were more effective inhibiting the mycelial growth than the derivatives with longest chains. The compounds **4** and **5**, with long alkyl chains and highest log *P* and volume values had little or almost no effect on the growth of the fungus. Therefore, a high degree of lipophilicity and biggest volume were not adequate for a good antifungal activity against *B. cinerea*. Other authors have also demonstrated that there is apparently an optimum hydrophobic balance for the antifungal properties of the molecules [15,18]. The following experiments were carried out only with the compounds **1** and **2** due to the fact that these are natural compounds.

### 2.3. Effect of compounds **1** and **2** on conidia germination and on ability of B. cinerea to colonize tomato leaves

**Figure 3:**
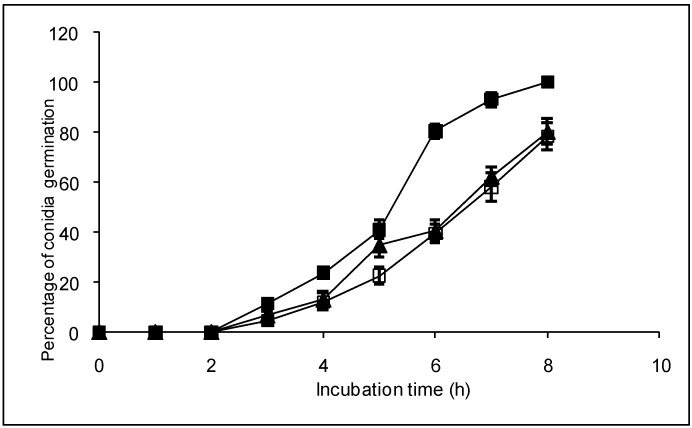
Effect of compounds **1** and **2** on conidia germination of *B. cinerea*. Control with methanol (■); compound **1** (▲) or **2** (□) at 60 μg mL^-1^. Compounds were added dissolved in methanol. Slides containing culture medium were inoculated with dry conidia, placed in a humid chamber, and incubated at 22 °C. Conidia germination was determined directly on the slides at intervals of 1 hour. Each point represents the mean of at least three independent experiments ± standard deviation.

Additionally, the effect of natural diterpenoids on the conidia germination and on the ability of the *B. cinerea* to colonize tomato leaves was evaluated. Both diterpenoids retarded conidia germination (Figure 3). 

**Figure 4:**
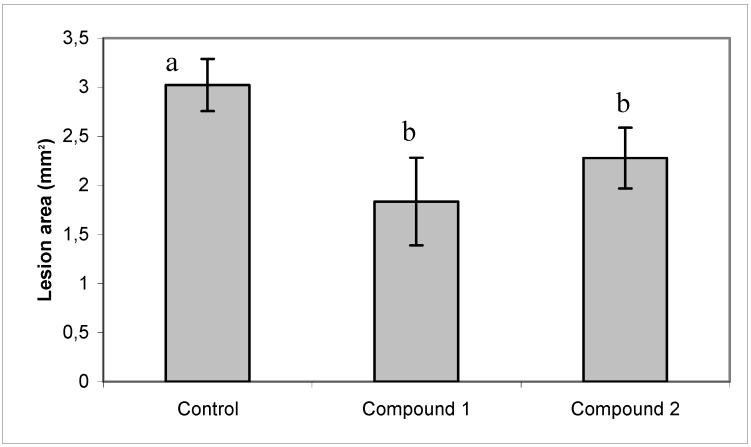
Effect of compounds **1** and **2** on ability of *B. cinerea* to colonize tomato leaves. The methanol solution (control) or compounds **1** or **2** at 60 μg mL^-1^ were spread on the surface of the leaves with a paintbrush. Five μL of a conidia suspension (10^5^ conidia mL^-1^) was inoculated on the upper side of the tomato leaves and leaves were incubated at 22 °C. After four days of incubation, the lesion area on tomato leaves was measured. Each bar represents the mean of at least three independent experiments ± standard deviation.Different letters indicate that the means are significantly different at *P* < 0.05.

After 8 h of incubation 100% of germination was reached in the control. Instead only 80% was observed in the presence of the compounds **1** and **2**. Diterpenoids did not produce morphological changes in the germ tube (data not shown). Also, a slight but significant protection to infection of tomato leaves by *B. cinerea* was observed in the presence of both diterpenoids (Figure 4). 

### 2.4. Mode of action compounds **1** and **2** on B. cinerea

**Figure 5:**
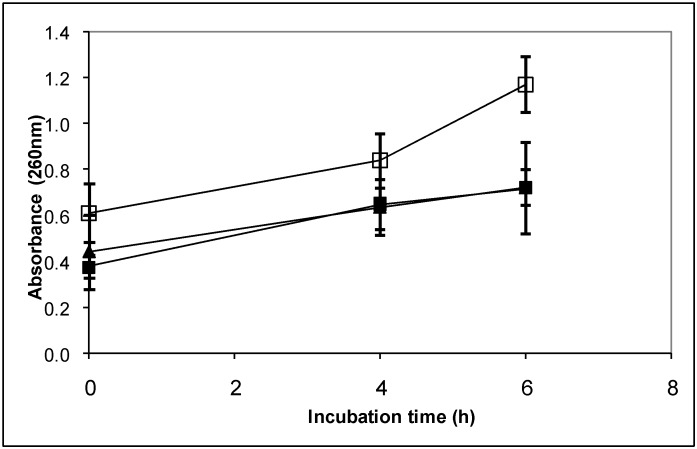
Cellular leakage of 260-nm-absorbing materials. Compounds **1** or **2** or methanol were added to cells washed with sodium phosphate buffer (5 mM, pH 7.0) and incubated at 22 ºC. At the specified times extracellular fractions were collected. Control with methanol (■), Compound **1** (▲) or compound **2** (□) at 60 μg mL^-1^. Each point represents the mean of two independent experiments ±standard deviation.

It has been reported that some diterpenoids interact with the fungal cytoplasmatic membrane causing its damage and, consequently, altering its permeability [6,7,8]. Therefore, the effect of diterpenoids **1** and **2** on the cytoplasmatic membrane of *B. cinerea* was analyzed determining the efflux of cellular components which absorb at 260 nm from mycelium treated with compounds **1** and **2** (Figure 5). 260 nm-absorbing components represent primarily nucleotides of which uracil-containing compounds exhibit the strongest absorbance [19].

Figure 5 shows that in the presence methanol or compound **1**, a similar increase of the absorbance at 260 nm was produced. Instead, after four or six hours of incubation, a highest increase of the absorbance, compared with the controls, was observed when *B. cinerea* mycelium was incubated with compound **2** indicating that this compound produced leakage of compounds from the mycelium.

**Figure 6:**
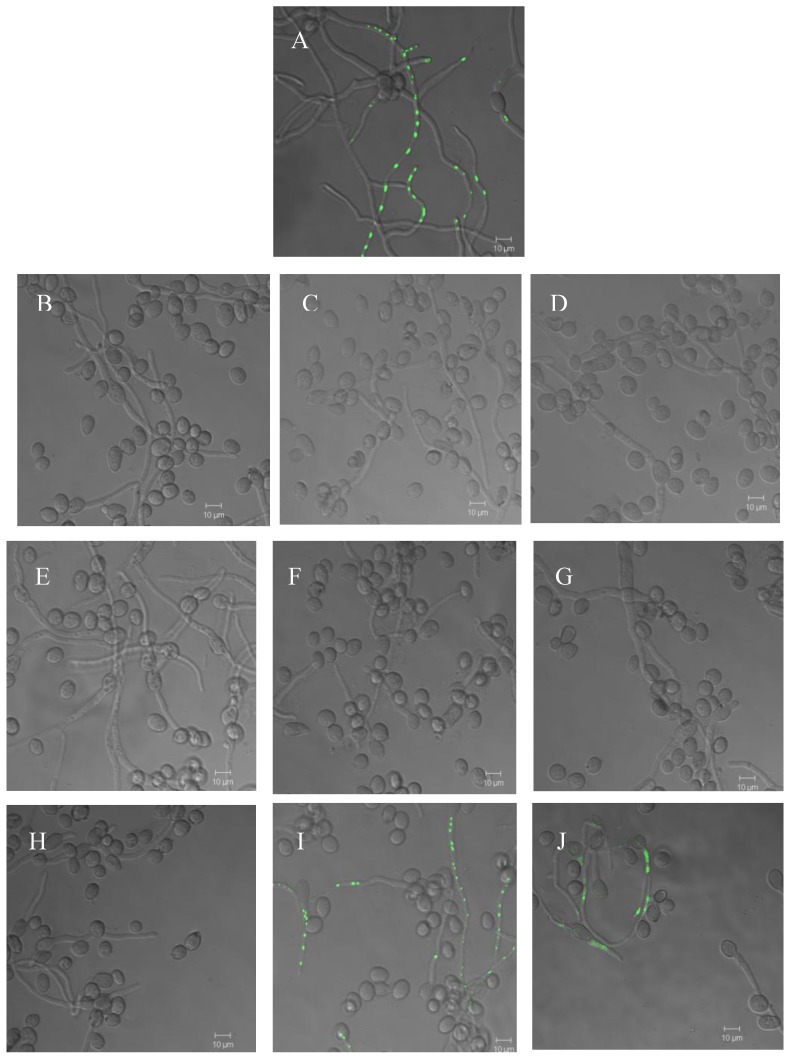
Effect of the diterpenoids on the *B. cinerea* cytoplasmatic membrane. Positive control, ethanol 70% (v/v) (A). *B. cinerea* in the presence of 8% (v/v) methanol (negative control) at 1 h (B), 4 h (C) and 6 h (D) of incubation; *B. cinerea* in the presence of 60 µg mL^-1^ salvic acid at 1 h (E), 4 h (F) and 6 h (G) of incubation; *B. cinerea* in the presence of 60 µg mL^-1^ acetylsalvic acid at 1 h (H), 4 h (I) and 6 h (J) of incubation. These figures are representatives of five independent experiments.

Additionally, the effect of the compounds **1** and **2** on *B. cinerea* cytoplasmatic membrane was analyzed using Sytox Green staining (Figure 6).

Ethanol was used as a positive control, because it causes dehydration of cellular membranes. When hyphae were treated with this compound, fluorescent nuclei were observed indicating alteration of the membrane (Figure 6A). In negative control, 8% methanol was used; in this case nuclei exhibited no fluorescence (Figures 6B-5D). When *B. cinerea* germinating conidia were treated with the diterpenoid **1**, after 1, 4 and 6 h of incubation, nuclei fluorescence was not observed which is an indication that membrane permeabilization to SYTOX Green did not occur (Figures 6E-6G). On the other hand, compound **2** produced alteration of the plasmatic membrane of *B. cinerea* after 4 or 6 h of incubation (Figures 6H-6J). These results were in accord with 260-nm absorbance assay.

**Figure 7:**
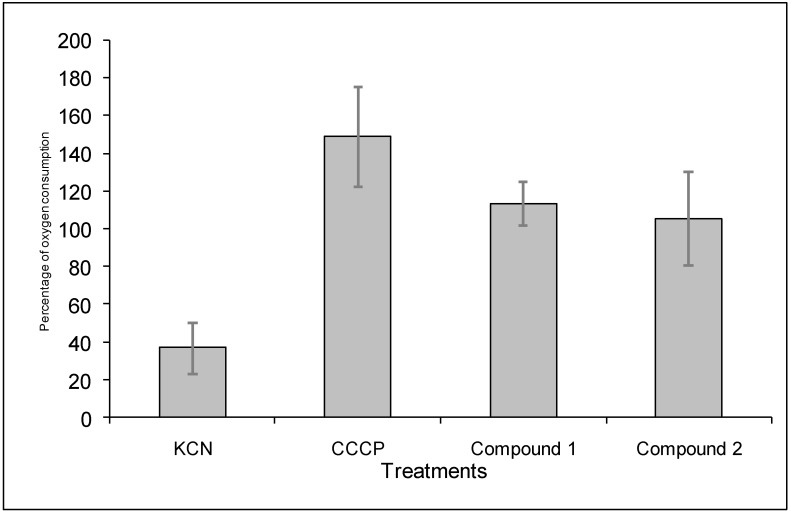
Effect of salvic acid and acetylsalvic acid on oxygen consumption of *B. cinerea* conidia. 10 μg mL^-1^ CCCP and 650 μg mL^-1^ KCN were used as control. Diterpenoids were added to conidia suspension dissolved in methanol at 160 μg mL^-1^. After eight min of incubation, the percentages of inhibition in the presence of the compounds relative to the basal oxygen consumption were calculated. Each bar represents the average of at least three independent experiments ± standard deviation.

Alternatively, it has been reported that some diterpenoids inhibit the respiratory chain in bacteria [10]. For this reason, the effect of the diterpenoids **1** and **2** on the oxygen consumption of germinating conidia of this fungus was also analyzed (Figure 7). KCN, an inhibitor of the respiratory chain, and CCCP, an uncoupler of the oxidative phosphorylation, were used as controls. 

In the presence of KCN, oxygen consumption decreased to 40%. KCN did not inhibit completely the oxygen consumption of *B. cinerea* conidia because this fungus contains a constitutive alternative oxidase [20]. The uncoupling compound increased oxygen consumption up to 150%. Finally, compounds **1** and **2** did not affect oxygen consumption at 160 μg ml^-1^. Similar results were obtained at lowest concentration of these compounds.

From the results obtained on the mode of action of compounds **1** and **2** can be concluded that these compounds exerted their effect by a different mechanism of action. Compound **1** did not affect cytoplasmic membrane or respiratory chain. Instead compound **2** altered the cytoplasmic membrane producing leakage of 260-nm absorbing compounds,

## 3. Conclusions

Diterpenoids salvic acid, acetylsalvic acid and propanoylsalvic acid presented higher antifungal activity against *B. cinerea* than butanoylsalvic acid. Diterpenoid isopentanoylsalvic acid did not show antifungal activity. Therefore, shortest side-chain diterpenoids were more effective than the derivatives with longer chains in the inhibition of *B. cinerea* mycelial growth. Salvic acid and acetylsalvic acid exerted their effect by a different mechanism of action. Salvic acid did not alter cytoplasmic membrane or respiratory chain inhibition. Instead, acetylsalvic acid affected the cytoplasmic membrane producing leakage of 260-nm absorbing compounds.

## 4. Experimental

### 4.1. General

The melting points (uncorrected) were determined on a Kofler hot-stage apparatus. IR spectra were recorded with a Bruker IFS 66v spectrophotometer. NMR spectra were acquired using a Bruker Avance RW- 400 spectrometer operating at 400.13 MHz (1h). Measurements were carried out at a probe temperature of 300k, using CDCl_3_ containing tetramethylsilane (TMS) as an internal standard. 

### 4.2. Chemicals

Malt extract was obtained from Cramer Co., Ltd. (Santiago, Chile). Yeast extract and DABCO (1,4-diazabicyclo[2. 2. 2]octane) were obtained from Sigma Chemical Co. (St. Louis, MO, USA). Agar was obtained from Difco laboratories (Detroit, MI, USA). Sytox Green was obtained from Molecular Probes (Eugene, OR, USA). Organic solvents and salts were obtained from Merck Química Chilena (Santiago, Chile).

### 4.3. Isolation of the natural diterpenoids salvic acid (**1**) and acetylsalvic acid (**2**) from E. salvia

Salvic acid (compound **1**) and acetylsalvic acid (compound **2**) were isolated from the resinous exudates of *E. salvia* leaves [11]. Voucher specimens (N° SGO-108833) were deposited in the Herbarium of the Museo Nacional de Historia Natural, Santiago, Chile. Whole leaves of fresh *E. salvia* were extracted by inmersion in dicloromethane for 15 s. The extract was separated by column cromathografic (silica gel) employing mixture of hexane-ethyl acetate, as has been described by Urzúa [11]. 

### 4.4. General procedure for the preparation of 7α-acyloxy-8(17)-labden-15-oic acids **3-5**

A mixture of compound **1** (100 mg, 3.1 x 10^5^ nmol), the appropriate acyl chloride (0.35 ml, 2.9 x 10^6^ nmol) and 4-dimethylaminopyridine (DMAP) (100 mg, 8.2 x 10^5^ nmol) in dichloromethane (20 ml) was stirred at room temperature for 72 h. The solution was washed with a 5% HCl and distilled water, then it was dried with anhydrous sodium sulfate and rotary evaporated. The crude product was purified by preparative thin-layer chromatography TLC, silicagel F_254_ (Merck Química Chilena, Santiago, Chile) and the purity was analyzed by TLC. The compound structure was determined by H^1^ NMR spectroscopy. With this procedure the following 7α-acyloxy derivatives were prepared: 7α-propanoyloxy-8(17)-labden-15-oic acid (Figure 1, compound **3**), colorless oil, 90 mg, 75% yield; 7α-butanoyloxy-8(17)-labden-15-oic acid (Figure 1, compound **4**), colorless oil, 89 mg, 73% yield and 7α-isopentanoyloxy-8(17)-labden-15-oic acid (Figure 1, compound **5**), colorless oil, 78 mg, 62% yield. 

### 4.5. Fungal Isolate and Culture Condition

In this study, the strain G29 of *B. cinerea* was used. This strain was originally isolated from a naturally infected grape (*Vitis vinifera*) [21] and was maintained on malt-yeast extract agar slants (2% (w/v) malt extract, 0.2% (w/v) yeast extract and 1.5% (w/v) agar) at 4 °C. The fungus was grown in the dark on malt-yeast extract agar medium [2% (w/v) malt extract, 0.2% (w/v) yeast extract and 1.5% (w/v) agar) or soft agar (2% (w/v) malt extract, 0.2% (w/v) yeast extract and 0.6 % (w/v) agar]. In the mechanism of action analysis, liquid minimum medium (KH2PO4 (1 g L-1), K2HPO4 (0.5 g L-1), MgSO4 x 7H2O (0.5 g L-1), KCl (0.5 g L-1), FeSO4 x 7H2O (0.01 g L^-1^) pH 6.5, 25 mol L^-1^ ammonium tartrate as a nitrogen source, and 1% (w/v) glucose as carbon source were used.

### 4.6. Effect of the compounds on the mycelial growth of B. cinerea in solid media

The fungitoxicity of diterpenoids and the commercial fungicide iprodione was assessed using the radial growth test on malt-yeast extract agar [22]. Diterpenoids and iprodione were dissolved in methanol and added at different final concentrations. The final methanol concentration was identical in control and treatment assays. Mycelial growth diameters were measured daily. After 72 hours of incubation the inhibition percentages relative to the control with methanol were calculated. Results were expressed as effective concentration (ED_50_) (the concentration that reduced mycelial growth by 50%) determined by regressing the inhibition of radial growth values (percent control) against the values of compounds concentration. Each experiment was done at least in triplicate.

### 4.7. Effect on the ability of B. cinerea to colonize tomato leaves.

Detached tomato (*Lycopersicon esculentum* cv. Roma) leaves were disinfected with 10% sodium hypochlorite, washed three times with sterile deionized water, and placed in Petri dishes containing water agar (1.5% w/v agar). The methanol solution or compounds **1** or **2** at 60 μg mL^-1^ were spread on the surface of the leaves with a paintbrush. This procedure was repeated three times. A suspension of conidia was made in Gamborg’s B5 medium (Duchefa BV, Haarlem, The Netherlands), supplemented with 10 mM sucrose and adjusted to 10 mM potassium phosphate (pH 6). A 5 μL amount of this conidia suspension (10^5^ conidia mL^-1^) was inoculated on the upper side of the tomato leaves. Petri dishes were incubated at 22 °C. After four days of incubation, the lesion area on tomato leaves was measured.

### 4.8. Effect on germination of B. cinerea conidia

Conidial germination assays were carried out on microscope slides coated with soft agar medium (2 mm thickness). Compounds **1** and **2** were added dissolved in methanol at a final concentration of 60 μg mL^-1^. Methanol was allowed to evaporate prior to inoculation. The slides were inoculated with dry conidia obtained from sporulated mycelia (1 week old), placed in a humid chamber (90% relative humidity), and incubated in the dark at 22 °C for 7 h. Conidial germination was determined directly on the slides at intervals of 1 hour. The percentage of germination was estimated by counting the number of germinated conidia in five microscope fields each containing approximately 40 conidia. Conidia were judged to have germinated when the germ tube length was equal to or greater than conidial diameter. Each experiment was done at least in triplicate.

### 4.9. Determination of mode of action of compounds 1 and 2

#### 4.9.1. Determination of the effect of the diterpenoids on the membrane integrity of *B. cinerea*

To analyze if compounds **1** and **2** produce alteration of the permeability of *B. cinerea* cytoplasmic membrane, cell leakage was determined measuring 260-nm-absorbing materials released to the medium [19]. Fifty mL Erlenmeyer flaks containing 5 mL of minimum liquid medium with 1% (w/v) glucose were inoculated with a conidia suspension (1x10^6^ conidia mL^-1^). Cultures were incubated at 22ºC and 180 rpm during two days. Micelia were washed two times with 500 μL of 5 mM sodium phosphate buffer pH 7.0 and incubated in stationary conditions at 22ºC in the presence of the compounds **1** and **2** at 60 μg mL^-1^ or methanol at the same concentrations as treatments. Samples were taken at intervals and spun at 8,000 *g* for 5 min in microcentrifuge tubes. Absorbance at 260 nm was determined in the supernatants. Results presented are the means of values from at least two independent assays.

The effect on permeability of the cytoplasmatic membrane was also determined using the SYTOX Green uptake assay [23]. *B. cinerea* conidia at a final concentration of 1x10^5^ conidia mL^-1^ were inoculated in 24-well plates (lined with 12-mm glass coverslips) containing 1 mL of liquid minimum medium. Cultures were incubated at 22ºC for 15 h to permit the germination of the conidia. After this time, liquid medium was removed and same medium with 70% (v/v) ethanol (positive control), 8% (v/v) methanol (negative control), or 60 μg mL^-1^ of compounds **1** or **2** was added to each well. The mixtures were incubated at 22 ºC for one, four and six hours in the case of diterpenoids and methanol or for 10 min when ethanol was used. *B. cinerea* hyphae adhered to glass coverslips were washed three times with liquid minimum medium and were stained with 50 nmol L^-1^ SYTOX Green. After 10 min of incubation, the hyphae were washed with minimum medium and glass coverslips containing hyphae were mounted in slides. For the assembly of the samples in the slides, 15 µL of DABCO (1,4-diazabicyclo[2.2.2]octane) was used. The fluorescence of *B. cinerea* hyphae stained with SYTOX Green was observed under a confocal microscope (Carl Zeiss LSM 510) at an excitation wavelength of 488nm and an emission wavelength of 540 nm. These experiments were done at least in triplicate.

#### 4.9.2. Determination of the effect of the diterpenoids on the oxygen consumption of *B. cinerea* conidia

Oxygen consumption was determined polarographically at 25 °C with a Hansatech oxygen electrode by using germinating conidia in a total volume of 1 mL. To obtain conidia in suspension, Murashige and Skoog´s basal medium at 4.4 g L^-1^ (Phytotechnology Laboratories, Lenexa, KS, USA) was added to Petri dishes containing conidia. The conidia were harvested by scraping with a sterile spatula. To eliminate mycelium, the suspension was filtered through glass wool. The conidia concentration was adjusted to 1x 10^7^ conidia mL^-1^ with minimum liquid media, in the presence of 2 % (w/v) glucose. Conidia were incubated for 2 hours at 22°C. The measurement of basal oxygen consumption was carried out for 2 min in the same minimum liquid medium. After this time, 10 μg mL^-1^ carbonyl cyanide *m*-chlorophenylhydrazone (CCCP), 650 μg mL^-1^ KCN or the diterpenoids **1** or **2**, dissolved in methanol at a concentration of 160 μg mL^-1^ were added. Oxygen consumption was determined for eight more minutes.

### 4.10. Determination of log P and Volume

Log *P* and the volume of diterpenoids were calculated using the HyperChem (TM) program (Hypercube, Inc., Gainesville, FL, USA).

### 4.11. Statistical analyses

All results obtained from at least three independent experiments were expressed as mean ± SD. Significant differences were determined using a one way analysis of variance (Genstat 5, Realease 4.1). Means were separated using the least significant difference test (*P*<0.05).
